# Transcatheter aortic valve implantation in Uppsala University Hospital 2009–2023: outcomes and temporal trends

**DOI:** 10.48101/ujms.v130.10999

**Published:** 2025-02-28

**Authors:** Elisa de Wilde, Leonardo Olivetti, Stefan James, Christina Christersson, Sergio Buccheri, Rickard Lindblom, Azad Amin, Giovanna Sarno

**Affiliations:** a Department of Medical Sciences, Uppsala University, Uppsala, Sweden; b Uppsala Clinical Research Center, Uppsala, Sweden; c Department of Earth Sciences, Uppsala University, Uppsala, Sweden; d Centre of Natural Hazards and Disaster Science (CNDS), Uppsala University, Uppsala, Sweden; e Swedish Centre for Impacts of Climate Extremes (climes), Uppsala University, Uppsala, Sweden; f Department of Surgical Sciences, Uppsala University, Uppsala, Sweden; g Uppsala University Hospital, Uppsala, Sweden

**Keywords:** Aortic stenosis, TAVI, registry, mortality, survival analysis

## Abstract

**Background:**

In recent years, transcatheter aortic valve implantation (TAVI) has rapidly emerged as a key treatment option for aortic stenosis. TAVI has been performed at Uppsala University Hospital since 2009. Data on TAVI procedures have been collected in a nation-wide all-comer registry, the Swedish Transcatheter Cardiac Intervention Registry (SWENTRY). However, only limited analysis has been conducted on trends in short- and long-term outcomes of TAVI patients in Sweden.

**Methods:**

This registry-based cohort study aims to evaluate outcome trends and long-term prognosis in patients who underwent TAVI in a Swedish single center between 2009 and 2023. Survival outcomes were studied using the Kaplan–Meier method. Cox Proportional Hazards models were used to adjust for differences in patient characteristics over time.

**Results:**

In total, 1,741 TAVI procedures were performed between 2009 and 2023. Immediate procedural mortality and 1-year mortality averaged at 0.9 and 8.1%, respectively. Both procedural and long-term mortality showed a decreasing trend over time. Similar results were observed when controlling for comorbidities and age.

**Conclusions:**

Short-term outcomes and long-term prognosis have been constantly improving for patients undergoing TAVI within this study. Similar mortality and complication trends have been observed in other registry studies. These trends may be attributed to improvements in the quality of care, and the increased use of TAVI in lower risk patients.

## Introduction

Symptomatic aortic stenosis is the most common valve pathology requiring intervention in Europe (1). Without treatment, the prognosis is poor, with a survival rate of about 50% after 1 year (2). Since there is no effective medical therapy for severe aortic stenosis, valve replacement remains the primary treatment option (2, 3). This can be done using SAVR (surgical aortic valve replacement) or TAVI (transcatheter aortic valve implantation). TAVI is minimally invasive, making it suited to patients who are poor surgical candidates due to age and comorbidities (3–5). During this procedure a valve prosthesis is implanted with the help of a vascular catheter, commonly inserted in the femoral artery. While TAVI was initially only considered an option for patients with high surgical risk, there has been a trend towards also accepting patients with a lower risk profile (6–9). Yet, SAVR is still the first choice for the younger patients without severe comorbidities (10).

The number of TAVI procedures have increased at a fast pace in Europe and the United States over the last 10 years (6, 11). Registry studies show decreasing periprocedural mortality over time (6, 11–14), as well as a decrease in complications such as paravalvular leaks, vascular complications, and stroke (6, 11, 15–17). The need for a permanent pacemaker after TAVI remains one of the most common complications (6, 11). Improvements in outcomes are likely attributable to increased clinical experience, TAVI in lower risk patients, better methods for patient selection, and more advanced prostheses (15). Possible predictors of short-term mortality include advanced age, peripheral artery disease, highly symptomatic heart failure, and severe left ventricular dysfunction (16–18). Predictors of mortality after 1 to 2 years include atrial fibrillation, diabetes, chronic lung disease, kidney disease, and frailty (16, 19).

Data on TAVIs performed in Uppsala since 2009 have been collected in SWENTRY, a nationwide all-comer registry. Accounting for 15–20% of the nation’s TAVI surgeries in 2023, this center is a key contributor to the provision of TAVI care in Sweden (20). However, research on short- and long-term outcomes for Swedish patients remains limited. Updated data on risks and outcomes are crucial for informed treatment decisions. This study aims to provide a comprehensive overview of patient and procedure characteristics, trends in outcomes, and survival in these TAVI patients between 2009 and 2023.

## Materials and methods

This study was based on data extracted from SWENTRY, a nationwide all-comer TAVI registry supported by Uppsala Clinical Research Center. Data from SWENTRY were combined with data from the Swedish Population Register to obtain survival status. All patients who had TAVI in Uppsala University Hospital between May 2009 and December 2023 were included. No exclusion criteria were applied. Mortality data, along with procedure and patient characteristics, were available for nearly all patients. However, follow-up data on echocardiographic parameters, NT-proBNP and NYHA-class post-TAVI were limited to a smaller subgroup of patients residing in Uppsala County from 2009 to 2021. Patient characteristics and outcomes were reported in accordance with the VARC-2 criteria whenever possible (21).

The primary indication for TAVI was recorded in the registry as either aortic stenosis or aortic regurgitation. However, this variable was not consistently reported until 2020, resulting in many cases being labeled as ‘unknown’. As shown in Table 1, the number of known aortic regurgitation cases was 1.3%, making this study predominantly focused on aortic stenosis. Patients may also have both aortic stenosis and regurgitation and be labelled as regurgitation as the primary indication. Given the study objective of evaluating the Uppsala TAVI experience as a whole, as well as the risk of unreliable exclusion of regurgitation patients due to the extent of missing data, all patients were included in the study population.

**Table 1:** Characteristics at baseline, N = 1741.

Demographics						
Age (years)	79.4	± 6.8				
Sex						
Female	760	(43.7%)				
Male	981	(56.3%)				
BMI*	27.4	± 5.15				
Interquartile range		23.8–30.4				
Comorbidities	Yes		No		Unknown	
Hypertension	1452	(83.4%)	288	(16.5%)	1	(0.1%)
Overweight (BMI 25–30)	639	(36.7%)	1095	(62.9%)	7	(0.4%)
Obesity (BMI > 30)	473	(27.2%)	1261	(72.4%)	7	(0.4%)
Diabetes	451	(25.9%)	1289	(74.0%)	1	(0.1%)
Atrial fibrillation	548	(31.5%)	1192	(68.5%)	1	(0.1%)
Prior pacemaker	176	(10.1%)	1444	(82.9%)	121	(7.0%)
Previous stroke	189	(10.9%)	1551	(89.1%)	1	(0.1%)
Peripheral artery disease**	239	(13.7%)	1501	(86.2%)	1	(0.1%)
Previous PCI	551	(31.6%)	1189	(68.3%)	1	(0.1%)
Previous cardiac surgery	183	(10.5%)	1502	(86.3%)	56	(3.2%)
Chronic lung disease	278	(16.0%)	1462	(84.0%)	1	(0.1%)
Chronic kindey disease (stage 4–5)	84	(4.8%)	1653	(94.9%)	4	(0.2%)
Frailty**	313	(18.0%)	1358	(78.0%)	70	(4.0%)
Critical preoperative condition**	39	(2.2%)	1701	(97.7%)	1	(0.1%)
LV function						
Normal	254	(14.7%)				
Mildly reduced	166	(9.5%)				
Moderately reduced	1215	(69.8%)				
Severely reduced	104	(6.0%)				
Unknown	2	(0.1%)				
**NYHA**						
Class I	20	(1.1%)				
Class II	361	(20.7%)				
Class III	1263	(72.5%)				
Class IV	94	(5.4%)				
Unknown	3	(0.2%)				
**Anatomical factors**						
Porcelain aorta	63	(3.6%)	1634	(93.9%)	44	(2.5%)
Bicuspid valve	200	(11.5%)	1334	(76.6%)	207	(11.9%)
Aortic valve area - cm^2^ (± 1SD)	0.75	(± 0.18)				
TAVI indication						
Aortic stenosis	1063	(61.1%)				
Aortic regurgitation	23	(1.3%)				
Unknown	655	(37.6%)				

* N = 1733

** Definition included in Appendix.

A one-sided Wilcoxon’s Signed Ranks test was used to assess the significance of improvements in ordinal variables following TAVI, alongside survival analysis using the Kaplan–Meier method and Cox proportional hazard regressions. To determine if post-TAVI prognosis improved over time, regardless of age and comorbidities, we employed three different Cox models of increasing complexity, aiming to assess the impact of the TAVI procedure year on survival while accounting for changes in patient characteristics over time. The three models differ only in terms of the number of covariates added. Covariates were added incrementally between the models, to evaluate model stability and effect size consistency when assessing the impact of the variable of interest on survival. In the regression analysis, the procedure years 2009–2015 were grouped together as ≤ 2015 due to fewer procedures performed during this period. Hazard ratios were calculated with 95% confidence intervals. The assumption of proportional hazards for the year of procedure variable was tested using Schoenfeld’s residuals. Variable selection was based on previous literature (16–19). All analysis was done in R version 4.1.2.

## Results

### Patient characteristics

**Figure 1:**
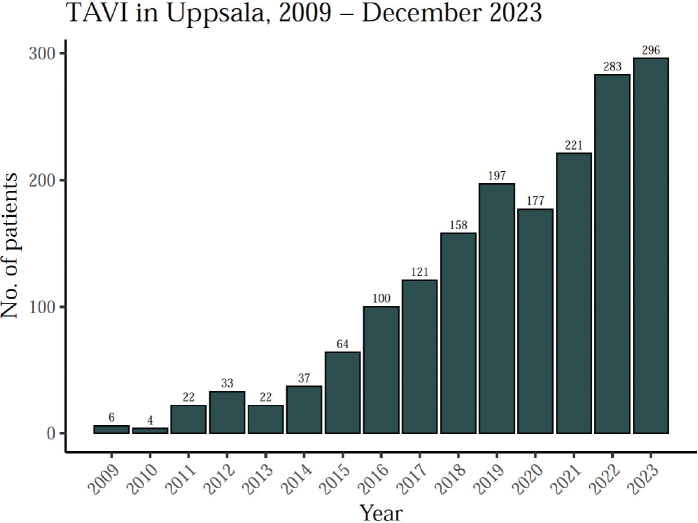
Number of TAVI procedures performed at Uppsala University Hospital, by year.

A total of 1,741 patients underwent TAVI between 2009 and December 2023. The number of TAVIs per year has steadily increased, exceeding 200 patients annually since 2019 (Figure 1). Patient characteristics are presented in Table 1. A detailed outlook on patient characteristics by year is included in the Appendix (Table A1). The study cohort had an average age of 79.4 years, comprising 47.3% women and 56.3% men. The study population spans a wide range of surgical risk levels. Hypertension and being overweight were the most prevalent comorbidities. Approximately 12% of patients presented with a bicuspid valve, and around 42% had undergone prior PCI or CABG.

### Procedural characteristics

Procedural characteristics are presented in Table 2. The most common access site was femoral. Predilatation of the stenotic valve with a balloon was performed in 71.7% of TAVIs, and postdilatation in 21.9%. Self-expanding prostheses, such as Evolut R or Pro, were most frequently used. Over time, valve-in-valve procedures have become more prevalent, accounting for 4.5% of total TAVI procedures.

**Table 2:** Procedural characteristics, N = 1741.

	N	Percentage
**Access**		
Transfemoral	1624	(93.3%)
Subclavian	110	(6.3%)
Transapical	2	(0.1%)
Direct aortic	5	(0.3%)
**Prosthesis model**		
Edwards Sapien 3 and 3 Ultra	175	(10.0%)
Medtronic CoreValve	158	(9.1%)
Medtronic Evolut FX, PRO, R	1377	(79.1%)
Other	18	(1.0%)
Unknown	13	(0.7%)
**Pre- and postdilatation**		
Predilatation		
In Sapien Valves	18	(10.3%)
In Medtronic Valves*	1214	(80.9%)
Postdilatation	381	(21.9%)
Unknown postdilatation status	2	(0.1%)
**Valve in valve procedure**		
Valve in valve, unspecified prior surgery	7	(0.4%)
TAVI with prior TAVI	9	(0.5%)
TAVI with prior SAVR	63	(3.6%)
Unknown	10	(0.6%)

* Unknown predilatation status in 35 patients in this cathegory.

**Table 3:** Outcomes during hospitalization, N = 1741.

Outcome	Yes		No		Unknown	
Mortality						
Immediate procedural (N = 1736)	15	(0.9%)	1721	(99.1%)	-	
< 3 days or in-hospital (N = 1717)	40	(2.3%)	1677	(97.7%)	-	
One year (N = 1429)	116	(8.1%)	1313	(91.2%)	-	
Major vascular complication*	30	(1.7%)	1480	(85.0%)	231	(13.3%)
Cardiac tamponade	27	(1.6%)	1705	(97.9%)	9	(0.5%)
Life-threatening bleeding*	72	(4.1%)	1455	(83.6%)	214	(12.3%)
Conversion to open surgery	5	(0.3%)	1735	(99.7%)	1	(0.1%)
Valve embolization	0	(0.0%)	1725	(99.1%)	16	(0.9%)
Coronary obstruction*	2	(0.1%)	1738	(99.8%)	1	(0.1%)
Myocardial infarction*	9	(0.5%)	1718	(98.7%)	14	(0.8%)
Permanent pacemakcr implantation**	163	(9.4%)	1273	(73.1%)	305	(17.5%)
Arythmia	3	(0.2%)	1704	(97.9%)	34	(2.0%)
Stroke or TIA*	32	(1.8%)	1664	(95.6%)	45	(2.6%)
Acute kidney injury stage 2–3*	10	(0.6%)	1692	(97.2%)	39	(2.2%)
Infection (of any type, which required antibiotics)*	37	(2.1%)	1690	(97.1%)	14	(0.8%)
Residual moderate-severe regurgitation*	81	(4.7%)	1616	(92.8%)	44	(2.5%)
Need for reintervention within 6 months	4	(0.2%)	1547	(88.9%)	190	(10.9%)
Successful procedure*	1679	(96.4%)	62	(3.6%)	0	(0.0%)
Length of hospital stay						
Median (days)	3					
Interquartile range (days)	2–6					

* Definition included in Appendix.

** Excluding patients with pacemaker before TAVI.

### Procedural outcomes: Mortality

**Figure 2:**
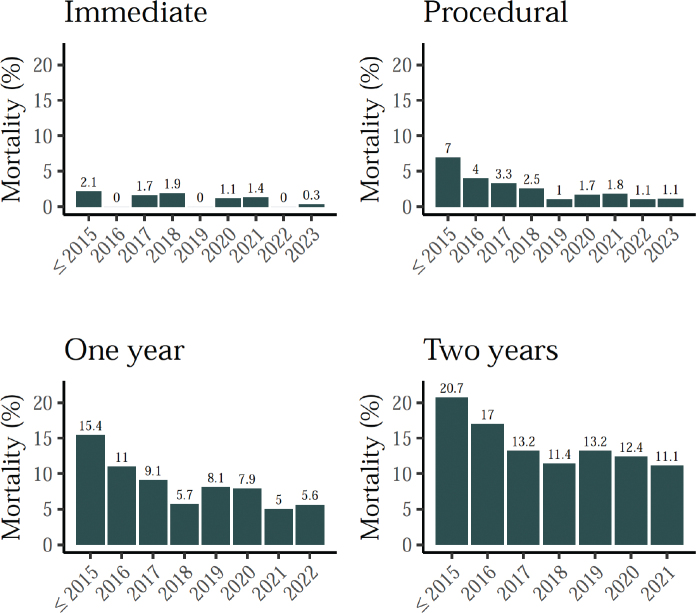
TAVI mortality rates, by year.

Immediate procedural mortality (within 72 h) and procedural mortality (within 30 days or in-hospital) were 0.9 and 2.3%, respectively. Both show a decreasing trend over time (Figure 2). Similar trends can be observed for one- or two-year mortality. For patients that had TAVI in 2020, almost 88% were alive 2 years after the procedure.

The improving trend in survival after TAVI is also apparent in the Kaplan–Meier analysis (Figure 5), and further supported by the Cox Proportional Hazards regression models (Figure 6). The hazard ratio of the year of procedure variable being below 1 (HR: 0.90) suggests that patients undergoing TAVI in more recent years generally experience better survival outcomes. This result maintains significance even when adjusting for comorbidities and age, as indicated by the stable hazard ratio and low *P*-value (*P* < 0.001) in all three models.

### Procedural outcomes: Complications

**Figure 3:**
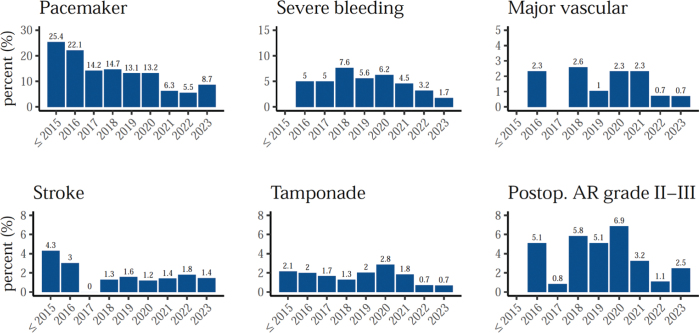
TAVI complication rates, by year.

The most common procedural complication was the need for a permanent pacemaker. As seen in Figure 3, this complication has decreased over time, from around 20% in the first years, to less than 10% in recent years. For more serious procedural complications such as myocardial infarction, major vascular complications, and stroke, the incidence was about 1–2%. These complications appear to have either slightly decreased over time or remained stably low.

### Procedural outcomes: Left ventricular function, New York Heart Association Class, NT-proBNP, walking test results

**Figure 4:**
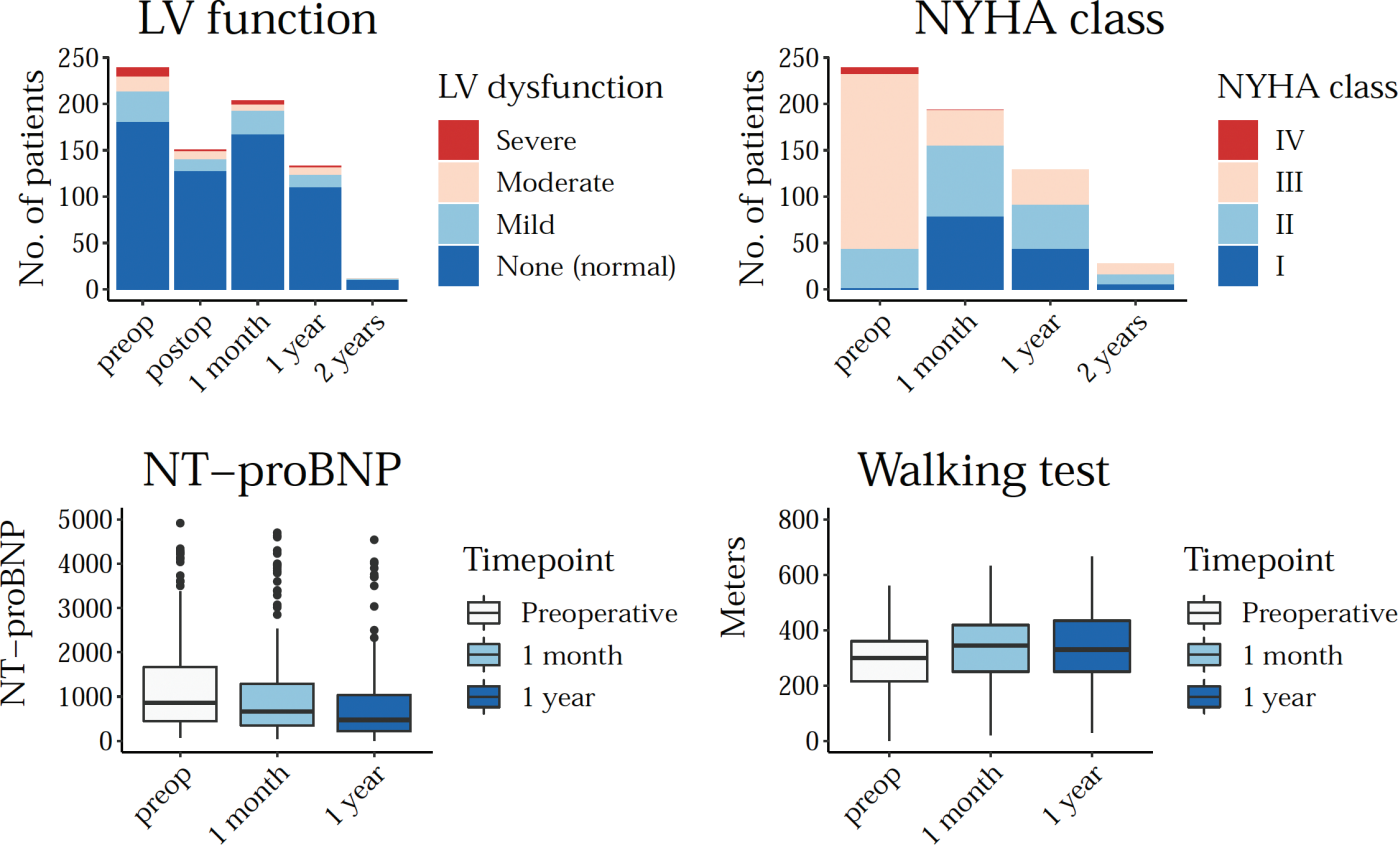
LV function, NYHA class, NT-proBNP, and walking test distance before and after TAVI.

For a subgroup of the population (residents of the Uppsala Region), follow-up data on left ventricular function, NYHA-class, walking test results, and NT-proBNP were available for the period 2009–2021. The median baseline NYHA-class was III, with a median improvement of one and a half NYHA classes at 30 days post-procedure compared to baseline, which was statistically significant (*P* < 0.05). Graphically, left ventricular function appeared to be improving after TAVI, and the walking test results also appeared to be improving over time, although these variables were not investigated with statistical testing. A summary of the difference between pre- and postoperative results for these four variables can be seen in Figure 4.

## Discussion

**Figure 5:**
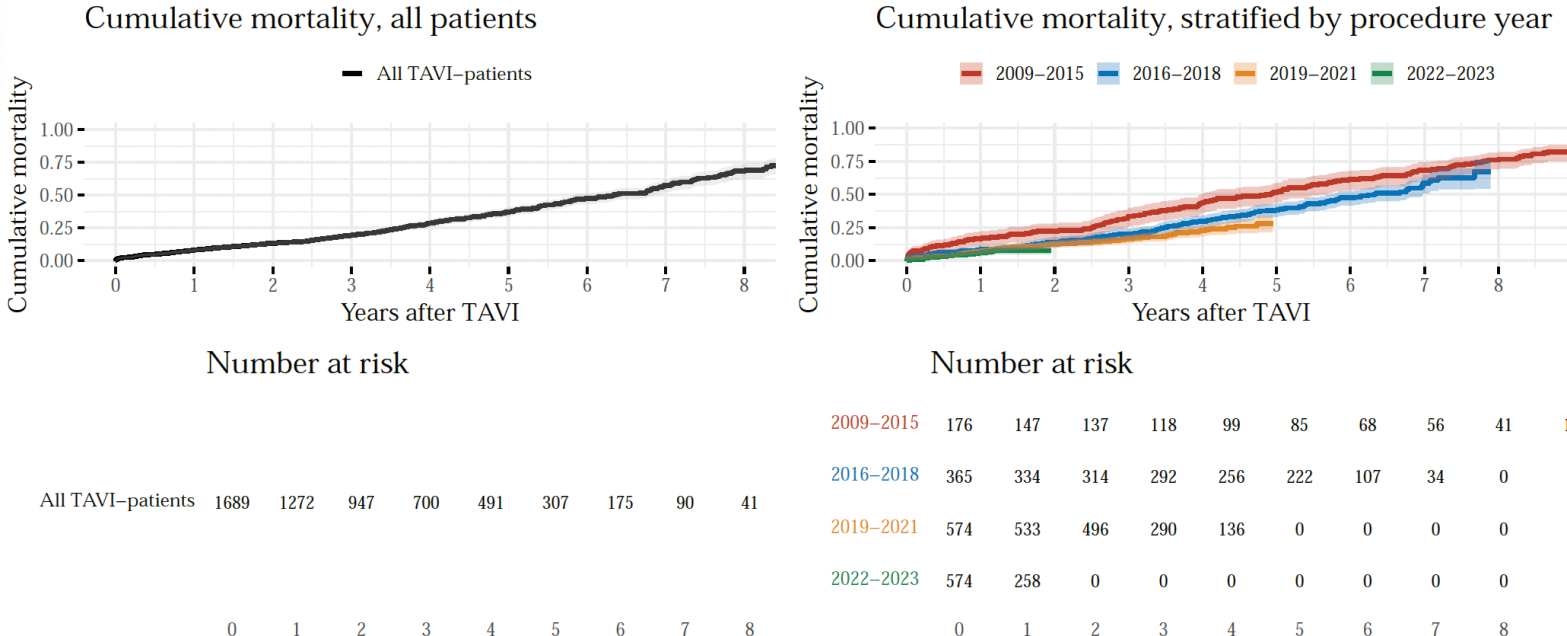
Inverse Kaplan–Meier curves, illustrating cumulative mortality over time in the years following TAVI. The left graph shows cumulative mortality for all patients, while the right graph presents cumulative mortality stratified by the procedure year.

**Figure 6:**
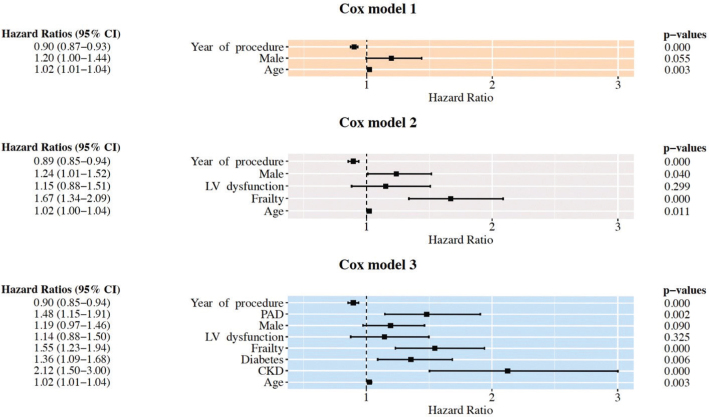
Cox Proportional Hazard models, showing survival after TAVI while controlling for comorbidities and age. Odds Ratios shown to the left, *P*-values to the right. Abbreviations: LV dysfunction = moderately to markedly decreased left ventricular function. CKD: chronic kidney disease stage 4–5. PAD: peripheral artery disease.

This all-comer retrospective registry study aimed to investigate trends in TAVI outcomes over time. The results show that the number of TAVI procedures has steadily increased (Figure 1), and post-TAVI survival has been improving throughout the study period (Figures 5–6). Additionally, the rate of severe complications appears to be stable or slightly decreasing, with the most apparent improvements in the decreasing rates of severe bleeding and severe postoperative regurgitation (Figure 3). Clinically, we also notice a decreased average time to discharge in recent years, from over a week up to 2017 to less than 3 days in the last few years. Unfortunately, data on endocarditis was not available in the registry. However, another study which merged the SWENTRY registry with the National Patient Registry (NPR) and the Swedish Registry on Infective Endocarditis (SRIE), found that the risk of endocarditis in the Swedish TAVI population was 1.4% in the first year after TAVI, and 0.8% per year thereafter (22).

The results of our study corroborate the findings of other registry studies on TAVI outcomes in the United States and Europe (7, 12), suggesting meaningful improvements in procedural outcomes over time and an increasingly central role for TAVI in the treatment of patients with aortic valve stenosis at lower surgical risk. Indeed, improved prognosis for TAVI patients may be partially attributable to the latter, as TAVI is being performed in lower-risk patients to a greater extent than before. However, the positive trend in survival persists even when controlling for age and comorbidities, suggesting additional reasons beyond patient characteristics. This points toward the possibility that outcomes may also have been improved due to quality-of-care factors, such as technical advancements in prostheses and implantation techniques, better patient selection, and increased clinical experience (15).

One of the main strengths of our study lies in the large study population, with complete survival data throughout the study period. Our real-world population has a higher technical risk profile in comparison with more recent randomized trials in lower risk profile patients (7, 23), including a sizeable number of patients with bicuspid aortic valve, porcelain aorta, valve in valve procedures, previous stroke and CABG, moderate to severe reduction of the LV function, peripheral artery disease, and patients requiring alternative vascular accesses, thus offering insights about outcomes also for patients with higher technical risk profiles.

Moreover, a large single center study offers a unique perspective on how procedural changes can be implemented within one center, and the direct effects of these changes. For instance, to reduce the need of pacemaker implantations after TAVI, the center adopted the Cusp Overlap technique in mid-2020. Starting from 2021, the implantation rate has seen a sharp decrease (Figure 3) likely attributable to this procedural improvement. Likewise, the rates of cardiac tamponade appear to have decreased since the implementation of left ventricular wire pacing at a similar time. This illustrates how meaningful clinical advancements can be achieved within a single center, impacting a substantial number of patients. Quality registries have a crucial role in monitoring the clinical practices and their impact on the population with the final aim to improve the clinical management and prognosis of patients.

However, it is also important to acknowledge the inherent limitations of a retrospective registry study. One limitation is the potential for unaccounted selection bias introduced by changes in the study population over time. Additionally, unknown sources of confounding attributable to baseline characteristics or other pre-procedural information not recorded in the registry may have influenced the results. Lastly, as a single-center study, the generalizability of the results beyond the studied population may be limited, even though Uppsala University Hospital accounts for a sizeable part of all TAVI surgeries performed in Sweden (20).

Moving forward, we hope that our study may contribute to the quality-of-care for TAVI patients in Sweden and beyond. Nevertheless, further research is needed to establish whether the results of this study reflect the outcomes of TAVI procedures in other Swedish hospitals and are generalizable to other countries. Moreover, it would be of interest to analyze stratified outcomes for patient subgroups with less prevalent characteristics, such as very high age or uncommon risk factors. Although these subgroups could not be separately evaluated in this study due to the limited sample size, future research with larger cohorts could provide valuable insights.

## Appendix

### Definitions

**Chronic kidney injury**. eGFR 15–29 (stage 4) or eGFR < 15 (stage 5).

**Frailty:** VARC-2 definition. Slowness, weakness, exhaustion, wasting and malnutrition, poor endurance and inactivity, loss of independence.

**Recent myocardial infarction:** Myocardial infarction less than 3 months before TAVI.

**Peripheral artery disease:** One or several of the following: Claudication, carotid stenosis > 50%, previous or planned surgery on the abdominal aorta, extremity arteries or carotid arteries.

**Critical preoperative condition:** One or several of the following: ventricular tachycardia, ventricular fibrillation, cardiopulmonary resuscitation, preoperative cardiac massage, preoperative ventilation, preoperative inotropy, use of intra-aortic balloon pump, or preoperative AKI with oliguria < 10 mL/h or anuria.

**Mortality:** VARC-2 definition. Immediate procedural mortality is procedure-related death < 72 h post-procedure. Procedural mortality is all-cause mortality within 30 days post-procedure, or during procedure hospitalisation if the postoperative length of stay exceeds 30 days.

**Successful procedure:** Correct positioning of a single prosthetic heart valve into the proper anatomical location, with absence of immediate procedural mortality and with good structural function of the prosthetic valve (Vmax < 3 m/s, mean valve gradient < 20 mmHg, no moderate to severe regurgitation).

**Life-threatening or disabling bleeding:** A bleeding requiring four or more transfusion units, or if Hb dropped with > 50 g/L periprocedurally, or if there was a treatment-requiring bleeding into the pericardium, causing a tamponade.

**Acute kidney injury stage 2–3:** Based on the AKIN classification. Stage 2 AKI was defined as an increase in serum creatinine of > 200% as compared to a preoperative baseline value. Stage 3 AKI was defined as an increase in creatinine of > 300% compared to baseline, or a postoperative creatinine of > 354 mmol/L with an acute postoperative increase of at least 44 mmol/L.

**Major vascular complication:** A major vascular complication was reported if a major vascular complication was reported in accordance with VARC-2, or if a life-threatening bleeding or death was reported together with either surgery-requiring damage to the aortic annulus, the need for acute vascular surgery or an unspecified procedural vascular complication.

**Stroke:** Sudden-onset neurological symptoms of stroke, with a duration of > 24 h.

**TIA:** Sudden-onset neurological symptoms with a duration of > 24 h.

**Coronary obstruction:** VARC-2 definition.

**Myocardial infarction:** VARC-2 definition.

**Valve embolization:** Valve embolization after prosthesis deployment.

**Infection:** Any infection during hospital stay that required treatment with antibiotics.

**Echo parameters:** Echo parameters were not reported according to the VARC criteria but were collected from echo reports in patients’ medical records.

**Table A1:** Characteristics at baseline, by year.

Procedure year	Number of patients	≤ 2015	2016	2017	2018	2019	2020	2021	2022	2023
		N = 188	100	121	158	197	177	221	283	296
Age (years)	Mean	80.3	78.9	80.3	79.8	79.1	78.8	79.4	79.7	78.6
	1SD	6.95	7.78	5.99	7.09	7.11	6.98	6.26	5.70	7.53
Sex	Female	80 (42.6%)	48 (48.0%)	69 (57.0%)	81 (51.3%)	87 (44.2%)	70 (39.5%)	93 (42.1%)	112 (39.6%)	120 (40.5%)
	Male	108 (57.4%)	52 (52.0%)	52 (43.0%)	77 (48.7%)	110 (55.8%)	107 (60.5%)	128 (57.9%)	171 (60.4%)	176 (59.5%)
BMI	Median	25.7	27.4	27.5	26.4	27.1	26.2	26.5	26.7	26.7
	IQR	23.0–29.7	24.4–31.9	23.6–31.2	24.3–30.4	24.1–30.6	23.7–30.4	24.1–29.9	24.2–30.0	23.7–30.2
Hypertension	Yes	119 (63.3%)	82 (82.0%)	100 (82.6%)	134 (84.8%)	160 (81.2%)	150 (84.7%)	196 (88.7%)	250 (88.3%)	261 (88.2%)
	No	69 (36.7%)	18 (18.0%)	21 (17.4%)	24 (15.2%)	37 (18.8%)	27 (15.3%)	25 (11.3%)	33 (11.7%)	31 (11.5%)
	Unknown									1 (0.3%)
Overweight (BMI 25–30)	Yes	66 (35.1%)	34 (34.0%)	36 (29.8%)	57 (36.1%)	71 (36.0%)	62 (35.0%)	87 (39.4%,)	115 (40.6%)	111 (37.5%)
	No	122 (64.9%)	66 (66.0%)	85 (70.2%)	100 (63.3%)	123 (62.4%)	112 (63.3%)	132 (59.7%)	164 (58.0%)	183 (61.8%)
	Unknown				1 (0.6%)	3 (1.5%)	3 (1.7%)	2 (0.9%)	4 (1.4%)	2 (0.7%)
Obesity (BMI > 30)	Yes	42 (22.3%)	34 (34.0%)	41 (33.9%)	42 (26.6%)	56 (28.4%)	47 (26.6%)	55 (24.9%)	71 (25.1%)	77 (26.0%)
	No	146 (77.7%)	66 (66.0%)	80 (66.1%)	115 (72.8%)	138 (70.1%)	127 (71.8%)	164 (74.2%)	208 ( 73.5%)	217 (73.3%)
	Unknown				1 (0.6%)	3 (1.5%)	3 (1.7%)	2 (0.9%)	4 (1.4%)	2 (0.7%)
Diabetes	Yes	41 (21.8%)	25 (25.0%)	29 (24.0%)	43 (27.2%)	45 (22.8%)	38 (21.5%)	63 (28.5%)	85 (30.0%)	82 (27.7%)
	No	147 (78.2%)	75 (75.0%)	92 (76.0%)	115 (72.8%)	152 (77.2%)	139 (78.5%)	158 (71.5%)	198 (70.0%)	213 (72.0%)
	Unknown									1 (0.3%)
Atrial fibrillation	Yes	50 (26.6%)	28 (28.0%)	42 (34.7%)	50 (31.6%)	63 (32.0%)	55 (31.1%)	75 (33.9%)	85 (30.0%)	100 (33.8%)
	No	138 (73.4%)	72 (72.0%)	79 (65.3%)	108 (68.4%)	134 (68.0%)	122 (68.9%)	146 (66.1%)	198 (70.0%)	195 (65.9%)
	Unknown									1 (0.3%)
Prior pacemaker	Yes	4 (2.1%)	14 (14.0%)	14 (11.6%)	15 (9.5%)	22 (11.2%)	24 (13.6%)	30 (13.6%)	28 (9.9%)	25 (8.4%)
	No	64 (34.0%)	86 (86.0%)	107 (88.4%)	143 (90.5%)	175 (88.8%)	153 (86.4%)	191 (84.1%)	255 (90.1%)	270 (91.2%)
	Unknown	120 (63.8%)								1 (0.3%)
Previous stroke	Yes	13 (6.9%)	16 (16.0%)	19 (15.7%)	21 (13.3%)	24 (12.2%)	26 (14.7%)	14 (6.3%)	33 (11.7%)	23 (7.8%)
	No	175 (93.1%)	84 (84.0%)	102 (84.3%)	137 (86.7%)	173 (87.8%)	151 (85.3%)	207 (93.7%)	250 (88.3%)	272 (91.9%)
	Unknown									1 (0.3%)
Peripheral artery disease	Yes	23 (12.2%)	18 (18.0%)	19 (15.7%)	36 (22.8%)	18 (9.1%)	33 (18.6%)	28 (12.7%)	30 (10.6%)	34 (11.5%)
	No	165 (87.8%)	82 (82.0%)	102 (84.3%)	122 (77.2%)	179 (90.9%)	144 (81.4%)	193 (87.3%)	253 (89.4%)	261 (88.2%)
	Unknown									1 (0.3%)
Previous PCI	Yes	60 (31.9%)	41 (41.0%)	38 (31.4%)	57 (36.1%)	55 (27.9%)	61 (34.5%)	66 (29.9%)	100 (35.3%)	73 (24.7%)
	No	128 (68.1%)	59 (59.0%)	83 (68.6%)	101 (63.9%)	142 (72.1%)	116 (65.5%)	155 (70.1%)	183 (64.7%)	222 (75.0%)
	Unknown									1 (0.3%)
Previous cardiac surgery	Yes	58 (30.9%)	18 (18.0%)	24 (19.8%)	20 (12.7%)	23 (11.7%)	16 (9.0%)	24 (10.9%)		
	No	130 (69.1%)	82 (82.0%)	97 (80.2%)	138 (87.3%)	174 (88.3%)	161 (91.0%)	191 (86.4%)	263 (92.9%)	266 (89.9%)
	Unknown							6 (2.7%)	20 (7.1%)	30 (10.1% )
chronic lung disease	Yes	41 (21.8%)	29 (29.0%)	30 (24.8%)	31 (19.6%)	26 (13.2%)	22 (12.4%)	29 (13.1%)	32 (11.3%)	38 (12.8%)
	No	147 (78.2%)	71 (71.0%)	91 (75.2%)	127 (80.4%)	171 (86.8%)	155 (87.6%)	192 (86.9%)	251 (88.7%)	257 (86.8%)
	Unknown									1 (0.3%)
Chronic kidney disease (stage 4–5)	Yes	12 (6.4%)	5 (5.0%)	7 (5.8%)	7 (4.4%)	8 (4.1%)	7 (4.0%)	10 (4.5%)	15 (5.3%)	13 (4.4%)
	No	176 (93.6%)	95 (95.0%)	114 (94.2%)	151 (95.6%)	188 (95.4%)	169 (95.5%)	211 (95.5%)	268 (94.7%)	281 (94.9%)
	Unknown					1 (0.5%)	1 (0.6%)			2 (0.7%)
Frailty	Yes	33 (17.6%)	19 (19.0%)	26 (21.5%)	48 (30.4%)	58 (29.4%)	45 (25.4%,)	33 (14.9%)	26 (9.2%)	25 (8.4%)
	No	86 (45.7%)	81 (81.0%)	95 (78.5%)	110 (69.6%)	139 (70.6%)	132 (74.6%)	188 (85.1%)	257 (90.8%)	270 (91.2%)
	Unknown	69 (36.7%)								1 (0.3%)
Critical preoperative condition	Yes	10 (5.3%)	2 (2.0%)	4 (2.5%)	3 (1.9%)	5 (2.5%)	5 (2.8%)	4 (1.8%)	4 (1.4%)	2 (0.7%)
	No	178 (94.7%)	98 (98.0%)	117 (96.7%)	155 (98.1%)	192 (97.5%)	172 (97.2%)	217 (98.2%)	279 (98.6%)	293 (99.0%)
	Unknown									1 (0.3%)
LV function	Normal	122 (64.9%)	75 (75.0%)	84 (69.4%)	112 (70.9%)	144 (73.1%)	119 (67.2%)	160 (72.4%)	213 (75.3%)	186 (62.8%)
	Mildly reduced	23 (12.2%)	12 (12.0%)	24 (19.8%)	22 (13.9%)	27 (13.7%)	29 (16.4%)	29 (13.1%)	32 (11.3%)	56 (18.9%)
	Moderately reduced	27 (14.4%)	7 (7.0%)	11 (9.1%)	19 (12.0%)	19 (9.6%)	15 (8.5%)	13 (5.9%)	21 (7.4%)	31 (11.5%)
	Severely reduced	16 (8.5%)	6 (6.0%)	2 (1.7%)	5 (3.2%)	7 (3.6%)	14 (7.9%)	19 (8.6%)	16 (5.7%)	19 (6.4%)
	Unknown								1 (0.4%)	1 (0.3%)
Porcelain aorta	Yes	14 (7.4%)	10 (10.0%)	6 (5.0%)	9 (5.7%)	8 (4.1%)	3 (1.7%)	5 (2.3%)	4 (1.4%)	4 (1.4%)
	No	131 (69.7%)	90 (90.0%)	115 (95.0%)	149 (94.3%)	189 (95.9%)	174 (98.3%)	216 (97.7%)	279 (98.6%)	291 (98.3%)
	Unknown	43 (22.9%)								1 (0.3%)
Bicuspid aortic valve	Yes		20 (20.0%)	14 (11.6%)	11 (7.0%)	28 (14.2%)	21 (11.9%)	23 (10.4%)	33 (11.7%)	50 (16.9%)
	No		67 (67.0%)	107 (88.4%)	146 (92.4%)	168 (85.3%)	156 (88.1%)	197 (89.1%)	249 (88.0%)	244 (82.4%)
	Unknown	188 (100.0%)	13 (13.0%)		1 (0.6%)			1 (0.5%)	1 (0.4%)	2 (0.7%)
